# Examination of blood samples using deep learning and mobile microscopy

**DOI:** 10.1186/s12859-022-04602-4

**Published:** 2022-02-11

**Authors:** Juliane Pfeil, Alina Nechyporenko, Marcus Frohme, Frank T. Hufert, Katja Schulze

**Affiliations:** 1 Molecular Biology and Functional Genomics, Technical University of Applied Sciences, Hochschulring 1, 15745 Wildau, Germany; 2 Kharkiv National University of Radio Electronics, Kharkiv, Ukraine; 3 Institute for Microbiology and Virology, Brandenburg Medical School Theodor Fontane, Neuruppin, Germany; 4 Oculyze GmbH, Mobile Microscopy and Computer Vision, Wildau, Germany

**Keywords:** Mobile microscopy, Blood cell detection, Machine learning, Deep learning, Instance segmentation

## Abstract

**Background:**

Microscopic examination of human blood samples is an excellent opportunity to assess general health status and diagnose diseases. Conventional blood tests are performed in medical laboratories by specialized professionals and are time and labor intensive. The development of a point-of-care system based on a mobile microscope and powerful algorithms would be beneficial for providing care directly at the patient's bedside. For this purpose human blood samples were visualized using a low-cost mobile microscope, an ocular camera and a smartphone. Training and optimisation of different deep learning methods for instance segmentation are used to detect and count the different blood cells. The accuracy of the results is assessed using quantitative and qualitative evaluation standards.

**Results:**

Instance segmentation models such as Mask R-CNN, Mask Scoring R-CNN, D2Det and YOLACT were trained and optimised for the detection and classification of all blood cell types. These networks were not designed to detect very small objects in large numbers, so extensive modifications were necessary. Thus, segmentation of all blood cell types and their classification was feasible with great accuracy: qualitatively evaluated, mean average precision of 0.57 and mean average recall of 0.61 are achieved for all blood cell types. Quantitatively, 93% of ground truth blood cells can be detected.

**Conclusions:**

Mobile blood testing as a point-of-care system can be performed with diagnostic accuracy using deep learning methods. In the future, this application could enable very fast, cheap, location- and knowledge-independent patient care.

## Introduction

The potential of mobile microscopy for diagnostic purposes in human and veterinary medicine has already been investigated using different setups and for many applications. The utilisation varies between the diagnosis of infectious diseases such as tuberculosis, the identification of parasites (malaria, helminth infections) and the detection of viruses or bacterial spores [1]. The basis are primarily constructions based on a special hardware and a smartphone. A differentiation can be made between lens based [2–4], lensless [5] or digital holographic microscopy [6, 7] and ptychographic systems [8]. The lens based systems consist of conventional microscopy components (lenses, objective, eyepiece) and their accuracy or resolution and the field of view are strongly dependent on the efficiency of the smartphone camera used. In lensless microscopy, the sample is usually placed very close to the camera sensor and illuminated vertically. To improve resolution, digital holographic microscopy not only illuminates the cells or objects to create a wave front, but also creates an inference pattern by generating a reference beam. With both techniques, the image must be reconstructed [5]. Overall for lensless and holographic systems commercial mobile systems are generally not available and the reconstruction of the images is algorithmically complex [6]. For ptychographic systems a reconstruction is still necessary but easier in comparison to the aforementioned construction.

The use of a mobile phone has the advantage that it can be used directly for image acquisition, storage and especially for evaluation. In addition, cloud resources can be accessed via Internet connection to perform particularly computationally intensive examinations in a timely manner and/or to contact a doctor or medical facility. The development and utilisation of reliable point-of-care (POC) diagnostic tools is of outstanding importance for the medical care of the population of infrastructural underdeveloped regions. The lack of specialized professionals and facilities makes the application of a low-cost smartphone-based mobile microscope particularly advantageous for timely and proper treatment [1].

In this context, blood testing is a simple but beneficial opportunity to assess the general state of health and detect various signs of disease. With the help of a microscopic blood examination a specialist can determine the number of red blood cells (RBC), white blood cells (WBC), platelets (PLT) and notice morphological changes. For example, a deficiency of RBC is an indicator of anemia, WBC provide information about a possible infection, the number of PLT is crucial for blood coagulation and morphological changes can provide information about sickle cell disease [9].

Many scientists have already worked on automated detection of different blood cells from microscopic images. Previous approaches use conventional image recognition for segmentation of different blood components [10]. These efforts are focused on methods such as thresholding [4, 11–15], watershed algorithm [11, 12], morphological filters [12, 14, 16], color-based conversion/segmentation [4, 12, 13, 15–17], histogram equalization [14], active contours [3, 11] and haar cascade [14]. The results might reach the desired accuracy, but these approaches are very error-prone towards minimal changes in image acquisition, such as contrast, exposure, resolution etc. [18]. In addition, they mostly focus on only one [3, 12, 14–17] or two [4, 10, 11] cell types and are rarely applied on a mobile device [13, 14]. Recent methods for image segmentation are increasingly based on machine learning (ML)/deep learning (DL) strategies due to their robustness, higher accuracy, flexible deployment and generalization ability [18–20]. A distinction can be made between simple object detection, semantic- and instance segmentation approaches [19, 20]. Instance segmentation allows an exact delineation of each cell and detection of its morphology. Therefore, it is a beneficial method to make particularly precise statements about the different blood cell types. Current research on blood cell segmentation of microscopic images focuses on networks such as YOLO [21] and region-based convolutional neural networks (R-CNN) [22] for object detection; U-Net [23], Seg-Net [24] and fully convolutional networks (FCN) [25] for semantic segmentation and Mask R-CNN [26] for instance segmentation. There is only one published example of researchers Alam and Islam [27] using the YOLO network to detect all blood cell types (RBC, WBC, PLT). For training, they use the Blood Cell Count Dataset (BCCD) [28], which provides stained, microscopic blood images and annotations. They achieve 96.09% accuracy for detection of RBC, 86.89% for WBC and 96.36% for PLT. However, their object segmentation approach does not allow accurate delineation of the cell membrane and they require K-nearest neighbour (KNN) and intersection over union (IoU) based post-processing as the PLT are often detected twice. The Faster R-CNN [29] approach is used to segment either the WBC [30] or RBC [31] only. In publications on semantic segmentation of blood cells, U-Net [32–34], Seg-Net [35] and FCN [31] are appropriately adapted to likewise segment only one [33, 34] to two [32, 35] cell types. There are very few publications that establish instance segmentation using Mask R-CNN for RBC and WBC [36] or only WBC [37] detection. Dhieb et al. [36] achieve 92% accuracy for detection of RBC and 96% for WBC and Fan et al. [37] achieve 99% average accuracy for segmentation masks of WBC.

In order to train the DL algorithms, different publicly available databases such as BCCD [27, 29, 30], acute lymphoblastic leukemia image database (ALL-IDB) [34, 35, 38] or self-made microscopic images [31, 32] are used. However, all of them were acquired with a laboratory microscope and high quality setups. Only one paper uses image data acquired with a smartphone camera, but also generated with an automated laboratory microscope [32]. Consequently, there is no DL-instance segmentation approach for all three blood cell types, which uses microscopic images from a mobile setup whose images can be generated cheaply and quickly, but cannot match the quality of images from publicly available databases or laboratory microscopes.

## Methods

### Sample preparation and microscopy

Donor for all blood samples used in this study is the first author. A small drop of capillary blood was obtained by fingerprick and was taken up with the middle of a cover slip. Waldeck's Testsimplets® [39] were used to stain the different blood cell types. The cover slip with the drop of blood is placed on the prestained area of the slide. After 10 to 15 min the slide was microscopically evaluated. For that the Bresser Erudit DLX microscope [40] with an objective with 60 × magnification and a numerical aperture of 0.85 was used [41], resulting in a resolution of 0.32 µm. The eyepiece provides an additional 10 × enlargement resulting in a total magnification of 600x. For image acquisition the microscopic ocular was removed and a digital eyepiece camera with 5 megapixels [42] was inserted. This setup produces an equivalent magnification of 600x. A commercially available smartphone (Xiaomi Mi A2 [43]) was connected via USB and color microscopic images with a resolution of 1440 × 1080 pixels were taken with the mobile app OTG view [44]. Before the annotation was started all images were cut to a resolution of 1000 × 1000 pixels and 96 dots per inch (dpi) to provide a square, standard size for different DL networks. One pixel corresponds to 0.1 µm. With the goal of increasing the robustness of the DL algorithms, the infrared (IR)/ultraviolet (UV) blocking filter of the ocular camera was removed for some image acquisitions. This resulted in artificially noisy and color-shifted images, which could also be generated naturally by mobile use at different locations. A rapid analysis and inexperience of the performing person could produce inaccurate images that are not perfectly focused and make it difficult to optimally set the lighting, which is also influenced by ambient parameters (artificial light indoors or sunlight outdoors).

### Dataset and image labelling

**Fig. 1:**
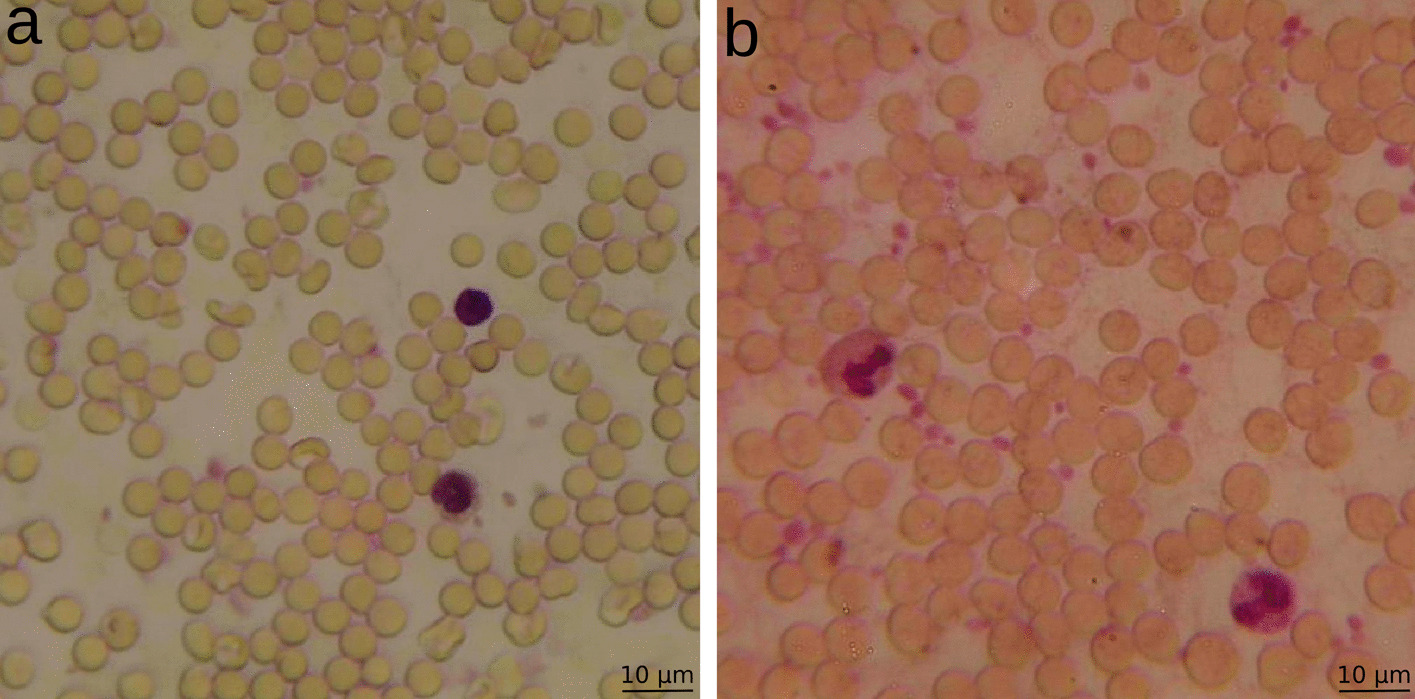
Microscopic images of stained blood samples at 600 × magnification. The preparation and microscopy of the blood samples were performed as described in the section "Sample preparation and microscopy". **a** High quality image, **b** low quality image. RBC are stained light red, WBC and PLT are stained dark purple

The dataset contains 40 microscopic pictures and each recording includes approx. 150–200 RBC, 2–4 WBC and 10–15 PLT. For training and validation a total of 5101 RBC, 71 WBC, 432 PLT were used. Images were prepared and annotated with the software labelme [45] and CVAT [46]. 30 images were taken with the normal camera setup (high quality images, Fig. 1a) and for ten images the color filter of the camera was removed (low quality images, Fig. 1b).

All RBC, WBC and PLT were labelled with polygon masks. 24 images were chosen for the training set (18 high quality and six low quality images) and eight images for the validation set (six high quality and two low quality images). A threefold cross validation [47] was performed with different splits of these 32 images resulting in three training and three validation sets. The remaining eight images (six high quality and two low quality images) were used for the test set containing 997 RBC, 14 WBC and 75 PLT and served to confirm the results.

### DL algorithms and training

For the targeted instance segmentation task of all blood cells, four different DL algorithms were implemented, optimised and the results evaluated. The well-known and extensively used Mask R-CNN [26] consists of 2 stages and is based on Faster R-CNN [29], which uses a region proposal network to predict bounding boxes for the different object classes. On top of this, Mask R-CNN predicts segmentation masks for the individual instances. Training and validation were done with the Mask R-CNN implementation of the toolbox MMDetection provided by Chen et al. [48]. Besides, Mask R-CNN is the predecessor of Mask Scoring R-CNN (MS R-CNN) [49]. This network significantly improves the accuracy of instance masks in the 2017 common objects in context (COCO) challenge [50]. The additional implementation of a network block termed as the MaskIoU head, which is trained with the quality of the predicted instance masks, improves the accuracy of the mask predictions. D2Det [51] is another two-stage detector based on the Faster R-CNN framework [29]. In addition, discriminative region of interest (RoI) pooling and dense local regression is applied for instance segmentation to improve accuracy and speed. YOLACT [52] is a one-stage framework for real time instance segmentation, which is characterized by an excellent inference speed, but therefore shows some drawbacks in segmentation accuracy. The network predicts a certain number of prototype masks that are generated by FCN [25] and calculates mask coefficients in parallel, which are multiplied together for each instance to create a linear combination of output masks. Training parameters for all frameworks have been customized for the existing graphics processing unit (GPU) infrastructure (GPU NVIDIA Tesla V100, DDR4-RAM 384 GB) and the task of blood cell instance segmentation. The following training parameters are common between adapted frameworks: training method stochastic gradient descent (SGD), momentum 0.9, backbone residual network with 101 layers (ResNet-101) [53] including feature pyramid network (FPN) [54] and augmentation methods such as resizing, random flips and change in hue etc. were applied. Furthermore, pre-trained weights based on ImageNet [55] and COCO datasets [50] were used for all frameworks. For YOLACT and D2Det the settings for Non-Maximum Suppression (NMS) were adjusted. In addition, anchor box size/scales were adapted for the detection of small objects and the number of possible detections per image respectively the number of trainable masks were increased. For YOLACT the number of predictions for NMS was changed from 200 to 400, the confidence threshold was decreased from 0.05 to 0.01 and the boxes threshold was modified from 0.5 to 0.1. For the anchor scales the configuration of YOLACT++ [56] was used. The number of masks was increased from 250 to 500 as well as the number of possible detections per image from 300 to 500. For D2Det the NMS threshold for the region proposal network (RPN) was decreased from 0.7 to 0.3 and the anchor scales were modified from 8 to 2. The number of detections per image were increased from 100 to 300. For MS R-CNN the number of possible detections per image were also increased in the MaskIoU head from 100 to 500. Other training parameters that vary between the different frameworks are shown in Table 1. The hyperparameters of the original networks and the modified values are listed. All networks were trained until no significant loss could be detected. The learning rate and batch size were chosen according to the GPU memory. The weight decay was applied according to the defaults in the available code from Github.

**Table 1:** Training parameters for Mask R-CNN, MS R-CNN, D2Det and YOLACT

Framework	# epoch		Learning rate		Batch size		Weight decay	Basis for pretrained weights
	Original	Modified	Original	Modified	Original	Modified		
Mask R-CNN	12	1000	0.02	0.02	2	2	0.0001	COCO [57]
MS R-CNN	3750*	1000*	0.02	0.00025	16	1	0.0001	ImageNet [58]
D2Det	24	3000	0.02	0.02	2	2	0.0001	COCO [59]
YOLACT	66.666*	1000–2000	0.0001	0.0001	8	2	0.0005	COCO [60]

^*^Calculated by: # epoch = number of iterations*batch size/number of training images

## Results

In this research work, different instance segmentation frameworks were modified, trained, optimised and their performance evaluated. The goal was to achieve the most accurate segmentation and classification of the blood components RBC, WBC and PLT on microscopic images generated with a mobile setup and a smartphone. Supported by a threefold cross validation, three different models were trained for each DL framework. The performance of these models is evaluated in the following sections. The results for the validation sets and the test set are presented visually (representation of predicted detections, including segmentation masks) and in addition the findings are described qualitatively (mean average precision (mAP) and mean average recall (mAR) of segmentation masks) and quantitatively (number of detected blood components).

### Visual results

**Fig. 2:**
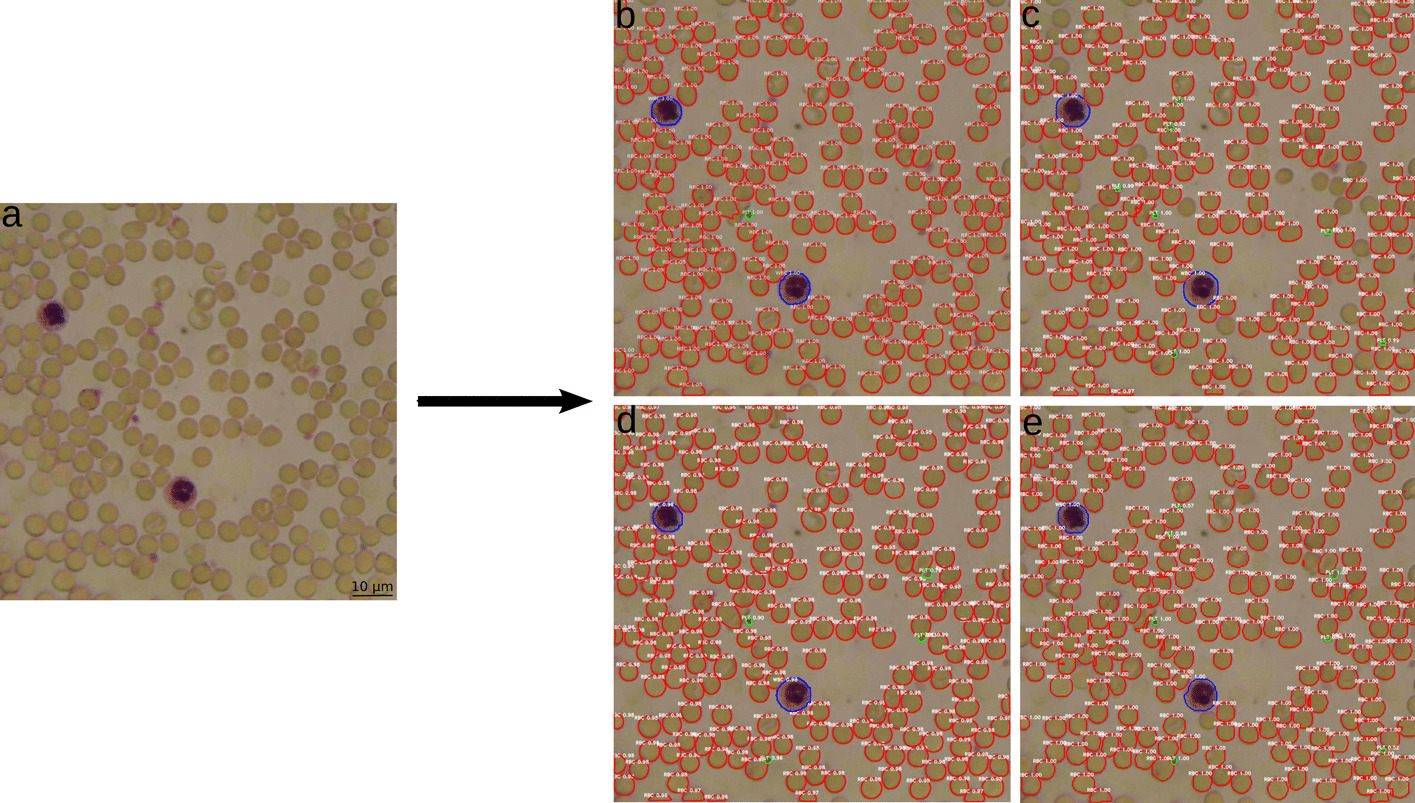
Visual representation of detected blood components (RBC—red, WBC—blue, PLT—green). **a** Original image** b** Mask R-CNN,** c** MS R-CNN,** d** D2Det and** e** YOLACT. Detections are shown at a high quality microscopic image of a stained blood sample at 600 × magnification from the validation set

**Fig. 3:**
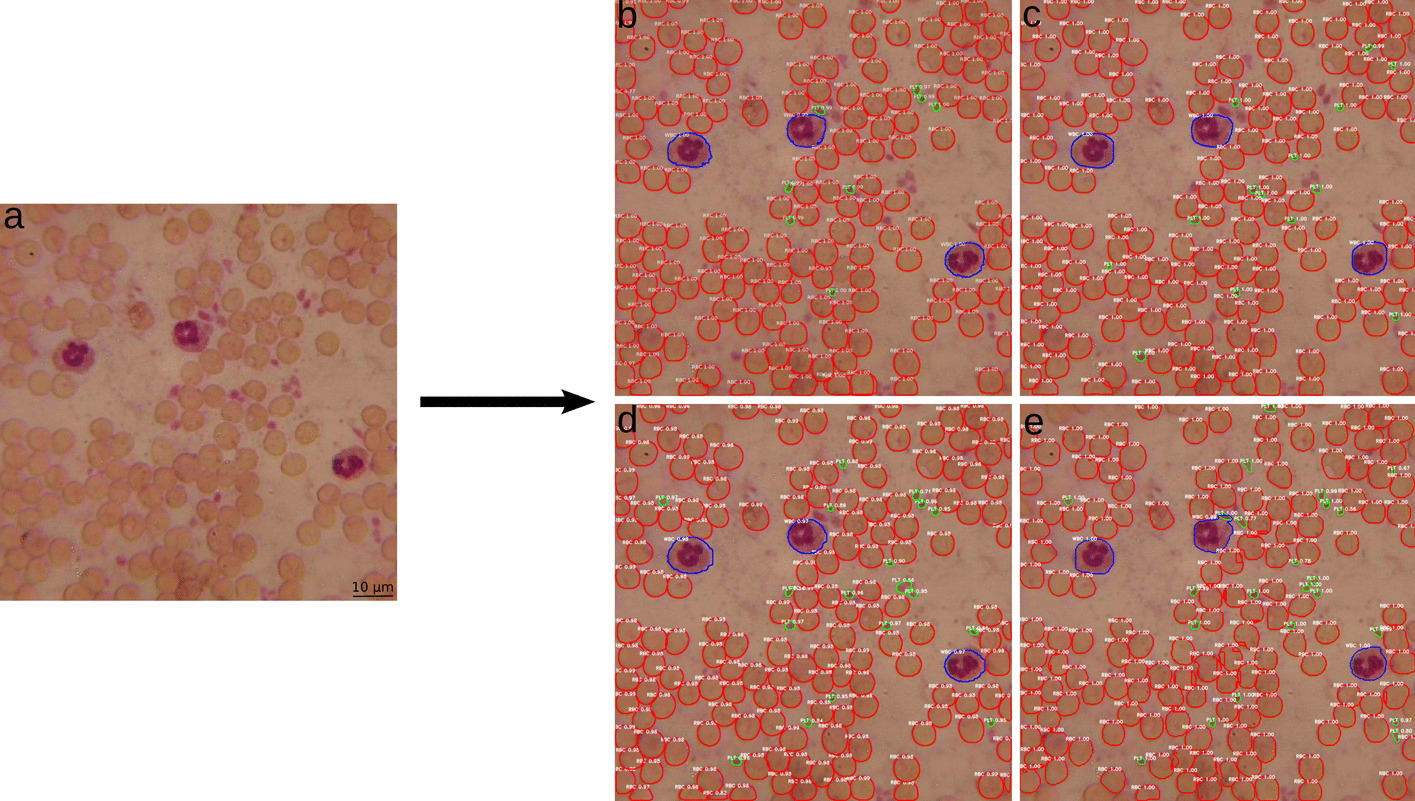
Visual representation of detected blood components (RBC—red, WBC—blue, PLT—green). **a** Original image** b** Mask R-CNN,** c** MS R-CNN,** d** D2Det and** e** YOLACT. Detections are shown at a low quality microscopic image of a stained blood sample at 600 × magnification from the validation set

Visually, the output has been unified for all trained frameworks. The predicted output masks were generated with an IoU threshold of 0.5. RBC are displayed in red, WBC in blue and PLT in green. Furthermore, each detection is labelled with the corresponding class and confidence score (quality of predicted mask and detected class). Figures 2 and 3 show the results of a cross validation output model for the frameworks Mask R-CNN, MS R-CNN, D2Det and YOLACT. In both Figures the detection results for all trained models basically do not show any difference between high and low quality images. All frameworks show a high confidence score for the different classes and accurate masks for WBC and PLT. The visual results for Mask R-CNN in Figs. 2b and 3b show several missing PLT detections. For trained MS R-CNN some RBC are not detected in Fig. 2c and some PLT are missing in Fig. 3c. D2Det failed to detect some PLT in Figs. 2d and 3d. Trained YOLACT shows slightly noisy masks for RBC and a few misdetections at the image edges in both pictures.

**Fig. 4:**
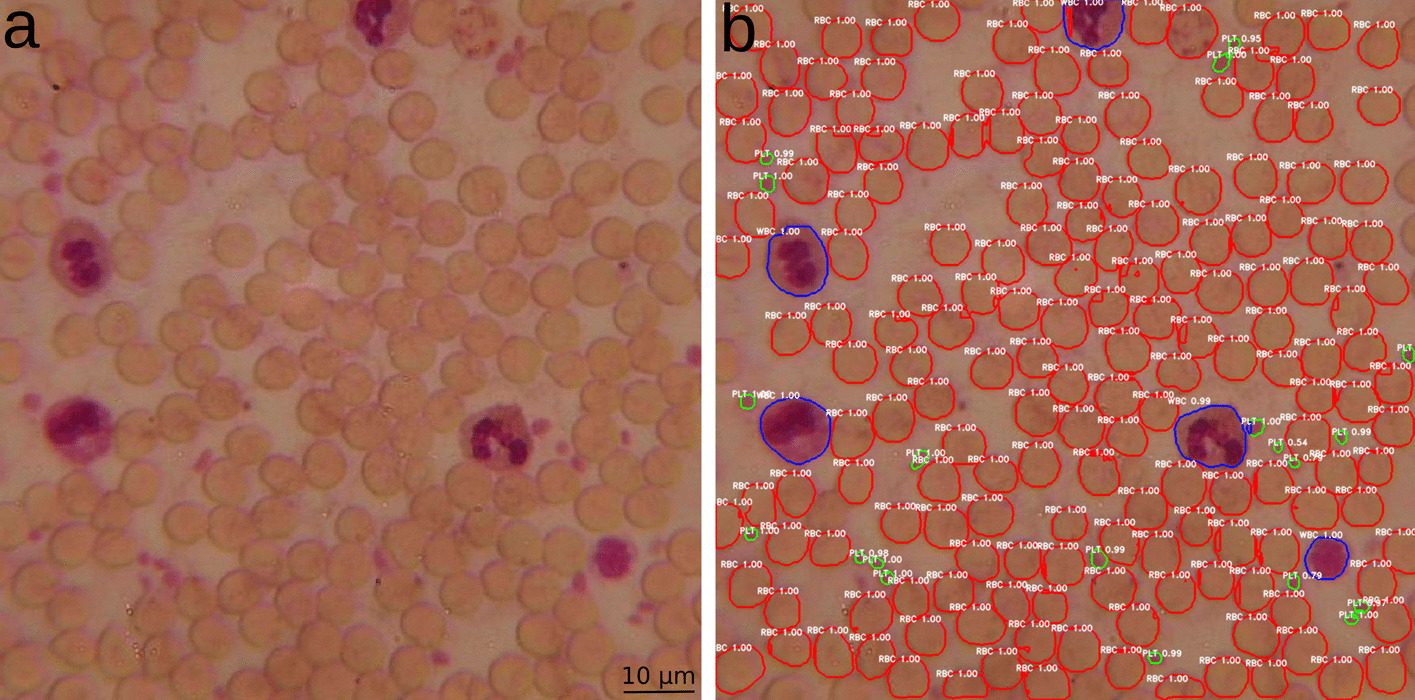
Visual representation of detected blood components (RBC—red, WBC—blue, PLT—green). **a** Original image **b **Trained YOLACT for unseen data. Detections are shown at a low quality microscopic image of a stained blood sample at 600 × magnification from the test set

In Fig. 4, a trained YOLACT model was used for a low quality image of the test set. The result also shows very good detection and segmentation output with no significant differences to results from the validation set.

### Qualitative results

For the assessment of the segmentation results, the mAP and the mAR were determined with IoU’s of 0.50 to 0.95 (step size 0.05) with a maximum of 100 detections aided by the COCO evaluation code [61]. In Table 2, the average of these values and their standard deviations (obtained by cross validation) for the trained models on the validation and the test set are shown and the best scoring outputs are marked in bold. mAP and mAR are mainly stable for the different models of cross validation, as indicated by the low standard deviation of mostly less than 0.05. Moreover, the different frameworks show the same tendencies for both parameters, e. g. MS R-CNN performs best for WBC in the validation set or YOLACT for PLT in the test set.

**Table 2:** mAP and mAR at IoU’s of 0.50 to 0.95 for the validation (v) and test set (t)

Framework	Dataset	Ø mAP ± σ				Ø mAR ± σ			
		All	RBC	WBC	PLT	All	RBC	WBC	PLT
Mask R-CNN	v	0.43 ± 0.02	0.46 ± 0.02	0.68 ± 0.06	0.15 ± 0.01	0.46 ± 0.02	0.48 ± 0.02	0.72 ± 0.06	0.18 ± 0.02
	t	0.47 ± 0.01	0.60 ± 0.01	0.65 ± 0.02	0.16 ± 0.02	0.50 ± 0.01	0.63 ± 0.01	0.67 ± 0.02	0.18 ± 0.02
MS R-CNN	v	**0.67** ± 0.01	0.53 ± 0.02	**0.91** ± 0.01	**0.56** ± 0.00	**0.69** ± 0.01	0.54 ± 0.02	**0.93** ± 0.01	**0.59** ± 0.02
	t	0.48 ± 0.02	0.58 ± 0.01	0.57 ± 0.06	**0.28** ± 0.01	0.53 ± 0.01	0.63 ± 0.01	0.61 ± 0.05	0.36 ± 0.02
D2Det	v	0.42 ± 0.04	**0.56** ± 0.02	0.46 ± 0.05	0.24 ± 0.07	0.47 ± 0.04	**0.60** ± 0.02	0.51 ± 0.05	0.30 ± 0.07
	t	0.44 ± 0.01	**0.61** ± 0.02	0.45 ± 0.01	0.25 ± 0.03	0.49 ± 0.01	**0.65** ± 0.02	0.50 ± 0.01	0.33 ± 0.04
YOLACT	v	0.45 ± 0.03	0.42 ± 0.02	0.71 ± 0.08	0.24 ± 0.05	0.49 ± 0.03	0.44 ± 0.02	0.74 ± 0.07	0.28 ± 0.09
	t	**0.50** ± 0.01	0.56 ± 0.02	**0.67** ± 0.02	**0.28** ± 0.03	**0.55** ± 0.00	0.60 ± 0.01	**0.69** ± 0.02	**0.37** ± 0.04

IoU’s of 0.50 to 0.95 with maximum of 100 detections for trained Mask R-CNN, MS R-CNN, D2Det and YOLACT for the validation and test set

**Fig. 5:**
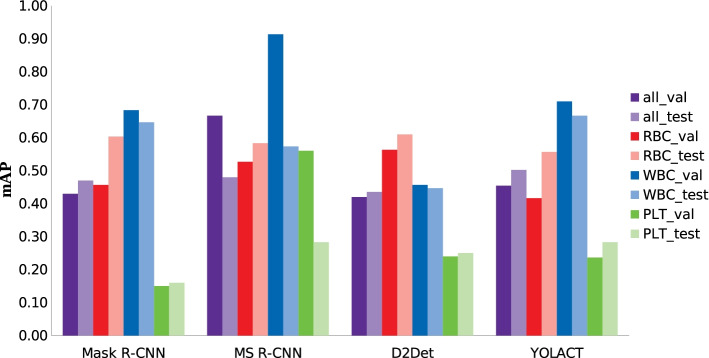
Bar chart of mAP values for the validation and test set for the different frameworks. The mAP at IoU’s of 0.50 to 0.95 for the average of all blood cells, RBC, WBC and PLT is shown for trained Mask R-CNN, MS R-CNN, D2Det and YOLACT

For a more straightforward visualisation, the mAP for both data sets is shown in Fig. 5 (the mAR performs equivalently and is therefore not shown).

The difference between validation and test set of each trained framework is mostly only 0.01 to 0.05 of the respective mAP. Larger deviations are only found in the values for the RBC for Mask R-CNN and YOLACT and for the WBC and PLT for MS R-CNN. Despite this, the results indicate a good generalisation ability of the models. The visual results from the previous section (Figs. 2 and 3) are also well reflected. The mAP of all models ranges between 0.42 and 0.61 for the RBC and between 0.45 and 0.91 for the WBC, confirming the high accuracy of masks and confidence scores for these classes. YOLACT achieves the lowest value for the RBC with a mAP of 0.42, visually also detectable by misdetections and noisy masks. The best performance for all blood components is achieved with the MS R-CNN model for the validation set and with YOLACT for the test set. With Mask R-CNN, the PLT in particular are poorly segmented and detected, which is also evident in Figs. 2a and 3a. However, the other trained models also achieve a good average output. Larger differences and the worst segmentation performance for all models is evident in the PLT. The reason for this is that basically smaller objects are more difficult to recognize and all frameworks were pre-trained and optimised for the COCO dataset. The smallest objects in this dataset correspond to 4% of the image size [50]. The PLT in the blood images, with an average size of 25 × 25 pixels, correspond to only 2.5% of the image size.

### Quantitative results

For the validation and the test set, the number of ground truth (GT) and predicted detections were also calculated for each class. In Table 3 the sum of the values for each cross validation for the trained models are shown and results closest to the GT are marked in bold. Mask R-CNN and D2Det provide the best results on quantitative accuracy for the RBC. For WBC, Mask R-CNN and MS R-CNN show nearly 100% detection accuracy for both data sets. But again, the weak performance of Mask R-CNN for the detection of PLT is significant.

**Table 3:** Number of detected blood cells (RBC, WBC, PLT) for the validation (v) and test set (t)

Framework	Dataset	∑ # detections		
		RBC	WBC	PLT
Mask R-CNN	v	3662	**54**	158
	t	**3058**	**41**	100
MS R-CNN	v	3636	**54**	256
	t	3059	40	193
D2Det	v	**3775**	55	242
	t	3089	40	184
YOLACT	v	3427	51	**311**
	t	2861	38	**231**

Trained Mask R-CNN, MS R-CNN, D2Det and YOLACT

GT numbers: *∑* validation sets RBC—3825, WBC—54, PLT—328

*∑* test set RBC—2991, WBC—42, PLT—225

**Fig. 6:**
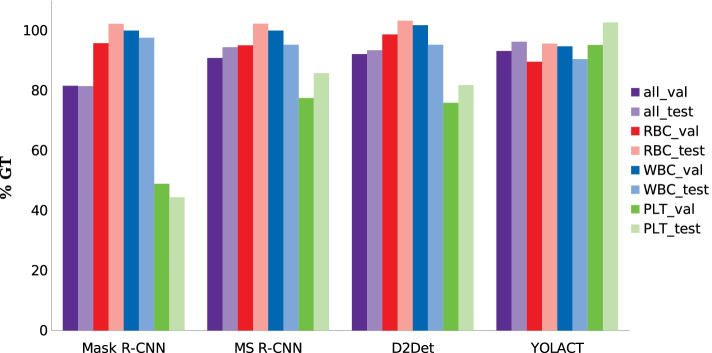
Bar chart of the proportion of detected blood components for the different frameworks. The percentage of the average of all detected blood cells, RBC, WBC and PLT of the GT is shown for trained Mask R-CNN, MS R-CNN, D2Det and YOLACT

For evaluation purposes, the predicted detections in % of the GT for the validation and test set and the different frameworks are shown in Fig. 6. The average performance for all blood components is shown as a separate bar in violet.

Similar to the qualitative results, only minor deviations between validation and test set are evident for the individual networks and the standard deviation is also mostly below 5%. Larger differences are only found for the PLT, as their small size and the associated detection difficulty can lead to greater fluctuations. However, these results once again confirm the generalisability of the models. On average, YOLACT performs best for all types of blood cells, followed by MS R-CNN, D2Det and Mask R-CNN. The detection results for RBC and WBC show very good scores for all models and frameworks with values above 90%. Only the detection performance of YOLACT for the RBC is a bit weaker in comparison to the other models. However, this is also clearly visible in the visual results where some RBC were not detected at the edges of the image. However, this model is able to detect most PLT compared to the other frameworks and achieves over 95% detection accuracy for both data sets.

## Discussion

In principle, all modified and trained models (Mask R-CNN, MS R-CNN, D2Det and YOLACT) achieve good to very good results in visual output, qualitatively and quantitatively, and could possibly be used for diagnostic purposes. The adjustment and optimisation of training parameters was absolutely necessary, as the original versions delivered very weak performance or were even unable to detect some blood cell types. To improve the relatively weak detection performance of Mask R-CNN (Ø 47% of GT for validation and test set) for PLT, further optimisation of the anchors to detect smaller objects should be performed. Furthermore, YOLACT shows very good results for the detection of WBC and PLT, but the performance for RBC is a bit weaker as these are often not detected at the image edges. Whether the detection of cells that are partly outside the field of view will be advantageous for diagnostic applications in the future has yet to be assessed. Otherwise, these cells could be ignored and would significantly improve the overall performance of the network. Pre-processing of the images with traditional image recognition algorithms such as thresholding, k-means clustering, contrast enhancement etc. are also conceivable to improve the overall performance.

The performance of the optimised instance segmentation models for the recognition of different blood components is also compared with existing similar research. Dhieb et al. [36] used the Mask R-CNN network as a basis for the instance segmentation of two blood components (no PLT) and achieved a quantitative accuracy of 92% for the detection of RBC and 96% for WBC. The detection accuracy of the models presented in this publication is up to 7% higher for RBC (Ø 99% for Mask R-CNN and MS R-CNN of GT for validation and test set). For WBC, Mask R-CNN, MS R-CNN and D2Det achieve a better result for the detection performance (Ø 99% for Mask R-CNN and D2Det, Ø 98% for MS R-CNN of GT for validation and test set). The training and validation set in this work contain in total only 71 WBC while Dhieb et al. [36] used a dataset of 150 images with 24.000 cells [62]. However, due to applied cross validation including calculated standard deviations, the comparison between the results of the validation and test set allow a statistically significant statement. The researchers in the aforementioned work did not perform a qualitative evaluation of the segmentation results. Alam and Islam [27] use YOLO as a basis for object detection of all blood components and achieve a quantitative accuracy for the detection of RBC of 96.09%, 86.89% for WBC and 96.36% for PLT. Again, the detection accuracy of the models in this publication is up to Ø 3% higher for RBC and Ø 13% higher for WBC. For PLT, only YOLACT achieves a higher detection performance of Ø 99% of GT for validation and test set. The authors achieved a mAP of 0.6236 as a qualitative measure, thus being slightly higher than the MS R-CNN presented (Ø 0.57 for validation and test set). However, Alam and Islam [27] use only object detection and not, as in this work, significantly more advanced instance segmentation. Both publications also use datasets [28, 61] with images taken at 1000 × magnification and standard microscopes, so that a significantly better resolution is available and the detection of the cells is consequently easier.

To further improve the presented models and increase their accuracy, the dataset should be enlarged to provide more images for training and validation. Furthermore, additional robustness can be generated by using other noise parameters to degrade the image quality, such as changing the illumination, using a camera with lower resolution etc.

## Conclusions

In this research work, the performance of DL-based instance segmentation algorithms for the detection of all blood cell types on microscopic images taken with a mobile microscope and a smartphone is thoroughly investigated. Training and optimisation of parameters for Mask R-CNN, MS R-CNN, D2Det and YOLACT network architectures were conducted. After examining the visual, qualitative and quantitative results, MS R-CNN performs in total best and achieved a Ø mAP of 0.57 and Ø mAR of 0.61 for the validation and test set for the segmentation of all blood cell types and was able to detect Ø 93% of all cells from both sets. All frameworks surpass their source versions in terms of visual output, qualitative (mAP, mAR) and quantitative results and their feasibility and effectiveness were demonstrated. Although some smartphone-based microscopes are already commercially available, the presented solution is innovative and its deployment is advantageous because mobile use of the optical system is already conceivable (lightweight, rechargeable microscope, digital eyepiece camera and mobile phone). A suitable smartphone application has to be developed for a location-independent evaluation of microscopic blood images. Future work will investigate the applicability of these algorithms in such an application allowing mobile analysis to be performed directly at the POC. In addition, further reduction in the size of the hardware will also increase mobility. Providing an autofocus and an automatic image capture are further adaptation options that enable easy utilisation regardless of location.

## Data Availability

The dataset generated and analysed during the current study is not publicly available because the research project was in cooperation with the company Oculyze GmbH, but is available from the corresponding author on reasonable request.

## Abbreviations

ALL-IDB1: Acute lymphoblastic leukemia image database
COCO: Common objects in context
DL: Deep learning
dpi: Dots per inch
FCN: Fully convolutional neural network
FPN: Feature pyramid network
GPU: Graphics processing unit
GT: Ground truth
IoU: Intersection over union
IR: Infrared
mAP: Mean average precision
mAR: Mean average recall
ML: Machine learning
MS R-CNN: Mask Scoring R-CNN
NMS: Non-Maximum Suppression
PLT: Platelets
POC: Point-of-care
RBC: Red blood cells
R-CNN: Region-based convolutional neural network
RoI: Region of interest
RPN: Region proposal network
ResNet-101: Residual network with 101 layers
SGD: Stochastic gradient descent
UV: Ultraviolet
WBC: White blood cells
